# HIV multidrug class resistance prediction with a time sliding anchor approach

**DOI:** 10.1093/bioadv/vbaf099

**Published:** 2025-05-15

**Authors:** Nurhan Arslan, Ralf Eggeling, Bernhard Reuter, Kristel Van Leathem, Marta Pingarilho, Perpétua Gomes, Anders Sönnerborg, Rolf Kaiser, Maurizio Zazzi, Nico Pfeifer

**Affiliations:** Methods in Medical Informatics, Department of Computer Science, University of Tuebingen, Tuebingen 72076, Germany; Institute for Bioinformatics and Medical Informatics (IBMI), University of Tuebingen, Tuebingen 72076, Germany; Methods in Medical Informatics, Department of Computer Science, University of Tuebingen, Tuebingen 72076, Germany; Institute for Bioinformatics and Medical Informatics (IBMI), University of Tuebingen, Tuebingen 72076, Germany; Methods in Medical Informatics, Department of Computer Science, University of Tuebingen, Tuebingen 72076, Germany; Institute for Bioinformatics and Medical Informatics (IBMI), University of Tuebingen, Tuebingen 72076, Germany; Laboratory of Clinical and Epidemiological Virology, Department of Microbiology, Immunology and Transplantation, Rega Institute for Medical Research, KU Leuven, Leuven 3000, Belgium; Global Health and Tropical Medicine, GHTM, Associate Laboratory in Translation and Innovation towards Global Health, LA-REAL, Instituto de Higiene e Medicina Tropical, IHMT, Universidade NOVA de Lisboa, Lisbon 1349-008, Portugal; Laboratório de Biologia Molecular, LMCBM, SPC, Unidade Local de Saúde Lisboa Ocidental, Hospital Egas Moniz, Caparica 2829-511, Portugal; Egas Moniz Center for Interdisciplinary Research (CiiEM), Egas Moniz School of Health and Science, Lisbon, Almada 1349-019, Portugal; Department of Medicine Huddinge, Karolinska University Hospital, Stockholm 14186, Sweden; Division of Infectious Diseases, Department of Clinical Microbiology, Karolinska Institutet, Stockholm 14152, Sweden; Institute of Virology, Faculty of Medicine, University Hospital Cologne, University of Cologne, Cologne 50935, Germany; Department of Medical Biotechnology, University of Siena, Siena 53100, Italy; Methods in Medical Informatics, Department of Computer Science, University of Tuebingen, Tuebingen 72076, Germany; Institute for Bioinformatics and Medical Informatics (IBMI), University of Tuebingen, Tuebingen 72076, Germany

## Abstract

**Motivation:**

The emergence of multidrug class resistance (MDR) in Human Immunodeficiency Virus (HIV) is a rare but significant challenge in antiretroviral therapy (ART). MDR, which may arise from prolonged drug exposure, treatment failures, or transmission of resistant strains, accelerates disease progression and poses particular challenges in resource-limited settings with restricted access to resistance testing and advanced therapies. Early prediction of future MDR development is important to inform therapeutic decisions and mitigate its occurrence.

**Results:**

In this study, we employ various machine learning classifiers to predict future resistance to all four major antiretroviral drug classes using features extracted from clinical HIV sequence data. We systematically explore several variations of the problem that differ in the pre-existing resistance level and the temporal gap between sample collection and observed MDR occurrence. Our models show the ability to predict multidrug class resistance even in the most challenging variations, albeit at a reduced accuracy. Feature importance analysis reveals that our models primarily utilize known drug resistance mutations for easier classification tasks, but rely on new mutations for the difficult task of distinguishing four class drug resistance from three class drug resistance.

**Availability and implementation:**

All analysis was performed using the Euresist Integrated DataBase (EIDB). Researchers wishing to reproduce, validate or extend these findings can request access to the latest EIDB release via the Euresist Network.

## 1 Introduction

Human immunodeficiency virus (HIV) remains a major global health concern despite receiving less public attention than other emerging viruses in recent years (Bekker *et al.* 2023). According to the World Health Organization (WHO), an estimated 39 million people worldwide live with the virus across different regions and populations (World Health Organization *et al.* 2021). In 2022, approximately 630 000 deaths were attributed to HIV-related illnesses, highlighting the ongoing and significant impact of the virus on global health (Owachi *et al.* 2024).

Advancements in medical treatments, especially antiretroviral therapy (ART), have been essential in managing the disease, reducing morbidity and mortality rates among HIV patients (Cohen *et al.* 2016, Peng *et al.* 2021). ART enables individuals to live longer and healthier and decreases the likelihood of HIV transmission to others (McNairy and El-Sadr 2014, Zazzi *et al.* 2018). Hence, HIV has been transformed from a fatal disease into a manageable chronic condition for many people. Despite advancements in treatment, there is no cure for HIV infection, requiring lifelong treatment.

ART targets HIV at different stages of its life cycle using medications classified into several distinct drug classes: nucleoside reverse transcriptase inhibitors (NRTIs), non-nucleoside reverse transcriptase inhibitors (NNRTIs), protease inhibitors (PIs), integrase inhibitors (INIs), fusion inhibitors, CCR5 antagonists, attachment inhibitors, and capsid inhibitors (Peng *et al.* 2021, Pirkl *et al.* 2023). Each class targets a specific stage of the virus’s replication process. ART uses mainly a three-drug therapy (Waters *et al.* 2021), which has been the standard since 1996, or two-drug combinations, which have been introduced more recently. A common feature of ART is to combine different drug classes to block virus replication at multiple stages and prevent the selection of drug resistance (Li *et al.* 2022). The primary objective of ART is to suppress HIV replication to undetectable levels by the commonly used lab assays, thus preventing disease progression (Hammer *et al.* 1997).

However, ART still faces several limitations, which contribute to treatment failure in HIV patients (Gupta-Wright *et al.* 2020). These challenges include adherence issues, drug toxicity (Bertrand *et al.* 2021), drug–drug and food–drug interactions in individuals taking other medications due to comorbidities (Khalilieh *et al.* 2020), resulting in incomplete virological suppression (Foka and Mufhandu 2023), and the emergence of drug resistance. In addition, infection may directly occur with drug-resistant HIV strains. Extensive resistance to multiple drugs and drug classes, referred to as multidrug class resistance (MDR), poses a critical problem, limiting treatment options and favoring disease progression (Datay *et al.* 2010, Cadosch *et al.* 2012). Although ART has slowed the development of resistance, MDR may occasionally occur, and a minority of people harbor an MDR virus due to prolonged drug exposure and multiple treatment failures (Feder *et al.* 2021, Peng *et al.* 2021, Pirkl *et al.* 2023).

While MDR loosely refers to resistance against more than one drug class, there is no commonly accepted definition of which and how many drug classes it pertains to. In this study, we define four drug class resistance (4CR) as resistance to the four most widely utilized drug classes, including NRTIs, NNRTIs, PIs, and INIs. The emergence of this form of MDR is rare in modern HIV treatment. However, when it occurs, it can be life-threatening, particularly in regions with limited access to advanced resistance testing and new antiretroviral drugs (Pirkl *et al.* 2023). Finding a new effective drug regimen for 4CR patients is challenging, prone to further failures with increasing resistance, and often requires the most recently developed high-cost drug classes, if available. Thus, preventing 4CR by choosing appropriate ART is the best approach to preserve treatment options. However, multiple patients and virus-related variables impact the risk of progression to the 4CR status, and reliable 4CR predictive models are essential.

Traditional rules-based approaches fall short in addressing this challenge, particularly due to the virus’s rapid mutation rate and the complexity of its evolutionary dynamics, patient-specific factors like treatment adherence, pre-existing resistant strains, and variability in treatment regimens (Pironti *et al.* 2017). These factors create class imbalances and inconsistencies in the sampling frequency and sample timing of virus sequence data, further complicating prediction. To overcome these challenges, machine learning (ML) might offer a more effective solution.

In this study, we apply various ML classifiers to predict the future occurrence of 4CR using features extracted from the clinical HIV sequence. Hereby, we vary the difficulty of the classification problem along two axes. First, we compile negative data sets exhibiting a varying degree of MDR prior to the first 4CR observation: from an easy case with zero drug class resistance in the negative to the most difficult one with existing resistance to up to NG classes. Second, we also vary the average time point at which the viral sample for predicting future 4CR has been extracted; the further in the past in relation to the actual 4CR occurrence, the more challenging the task becomes. We find that predicting 4CR is possible even in the most challenging case, albeit at a lower accuracy than in the easier settings. Finally, we also analyze feature importance to pinpoint the specific sequence features that most significantly influence the models’ predictions.

## 2 Methods

After first describing our data source and providing a concise summary of the extracted dataset, we then define drug resistance and drug class resistance as well as the classification setting. Finally, we present the full data preprocessing workflow, consisting of down-sampling nonresistant cases, reconstructing virus sequences, and sequence encoding.

### 2.1 Original data and initial filtering

The EuResist Integrated Data Base (EIDB) is one of the largest repositories of HIV genotypes and their corresponding clinical responses to ARTs. The EIDB is instrumental in advancing our understanding of HIV drug resistance, particularly in investigating the prevalence and clinical outcomes of 4CR viruses in patients. Additionally, it provides valuable insights into the factors that drive the development of 4CR. We use the June 2023 version of the EIDB for this study. All original databases that provide data to the EIDB received ethical approval within their respective countries. The EIDB stores data of 71 503 patients from 22 different database providers. We only use data from the patients who have measurements from raw sequences from three genes, reverse transcriptase (RT), protease (PR), and integrase (IN), drug resistance scores for each drug within the corresponding four drug classes, viral load measurements, and time points from each given measurement.

We filter the original data as follows. Patients with a single record are excluded, so only patients with at least two records are retained. Any sequence with missing gene information from the RT, PR, and IN regions is removed. Records with inconsistencies in sampling dates, such as discrepancies in the day, month, or year, are also filtered out. Additionally, sequences containing stop codons and degenerated codons translated into more than four amino acids are excluded from our analysis.

We construct a comprehensive patient profile by merging the relevant data from the EIDB for each patient. Each profile contains at least two hospital visits and includes the virus sequence sampling (raw sequence), the sampling date, sequence length, the specific gene regions, identified mutations, as well as drug resistance scores and corresponding resistance levels for each drug within the NRTI, NNRTI, PI, and INI drug classes, age, gender, and viral load (VL). These resistance details are obtained using the Stanford HIV Drug Resistance Interpretation System (see next section). The four drug classes contain a total of 17 drugs, with a comprehensive list available in Supplement S1.1. We exclude drug classes like coreceptor fusion inhibitors, gp120 blockers, capsid inhibitors, and CD4 binding monoclonal antibodies due to insufficient data in EIDB for these categories.

### 2.2 Multidrug class resistance

HIV drug resistance occurs when genetic mutations in the virus reduce the effectiveness of ART in inhibiting its replication. Within the context of ARTs, drug resistance is assessed based on specific mutations in the HIV genome. In EIDB, the stored resistance information is calculated using the Stanford HIV Drug Resistance Interpretation System, specifically version 8.9–1 of the algorithm that identifies mutation types and their associated resistance levels. This system quantifies drug resistance by assigning a score that reflects the degree of viral resistance to the specific antiretroviral drugs, ranging from susceptible (no resistance) to high-level resistance. Further details on the calculation of these resistance scores are provided in Supplement S1.

The standard sequence-based definition for INIs resistance is often insufficient, necessitating an additional approach for INIs. Unlike NRTIs, NNRTIs, and PIs, INIs are a relatively recent class of drugs and have demonstrated high efficacy. However, sequencing the IN gene of the virus is typically conducted only when resistance is suspected, resulting in limited sequence data available in our database. To compensate for this lack of INI data, we used an alternative definition for INI resistance in cases where IN sequencing data was unavailable. This definition suggests that therapies with INI can lead to resistance under specific conditions, as determined by VL measurements and the duration of INI drug treatment. Further details on this definition are provided in Supplement S1.

A virus is considered drug-resistant if it shows intermediate or high-level resistance to any specific drug. This method is called sequence-based drug resistance definition (Pirkl *et al.* 2023). If the virus displays intermediate or high-level resistance to at least one drug within a specific class, it is classified as drug-class resistant (CR) (Tang *et al.* 2012, Pirkl *et al.* 2023). As resistance extends to two or three drug classes, it is categorized as 2CR or 3CR, respectively. Furthermore, when resistance is detected across all the four key drug classes, NRTIs, NNRTIs, PIs, and INIs, the virus exhibits four-drug-class resistance (4CR). We provide an illustration that explains the different CR levels in Supplement S1.4.

### 2.3 Identification of positive and negative patients

We then categorize patients according to their infections with CR viruses, which can show resistance in none (0CR), one (1CR), two (2CR), three (3CR), or all four (4CR) drug classes. We reveal the following distribution: 54 475 patients harbored 0CR viruses, 11 346 had 1CR viruses, 10 616 had 2CR viruses, 3095 had 3CR viruses, and 108 had 4CR viruses.

Furthermore, we employ the VL-based resistance definition to determine if additional patients are infected with INI-resistant viruses in cases where the INI sequence data was unavailable. We identify 2600 patients with viruses resistant to the INI drug class, illustrated in Supplement S1.3.

This inclusion leads to 352 patients being infected with 4CR viruses. These patients represent the positive samples in our training dataset. The remaining 71 151 patients, who harbor non-4CR viruses spanning four CR levels from susceptible (0CR) to 3CR, represent the negative samples in the dataset. These results are illustrated in Supplement S1.5.

### 2.4 Time to MDR occurrence

We aim to identify the time it took for 4CR to develop. To this end, we use the first 4CR detection date as a reference point for each patient and calculate the time of all other records for the patient relative to the 4CR observation. We observe that patients with 4CR viruses often have irregular and infrequent virus sequencing during the first 10 years of treatment, indicating potential non-adherence. However, in the 2 to 3 years preceding the initial detection of 4CR, there is a significant increase in virus sequencing, with shorter intervals between samples.

### 2.5 MDR classification task

In our classification task, we use patient virus sequences as input data to predict whether the corresponding viruses will develop 4CR, with the outcome being either yes or no. We assess how far in advance 4CR prediction can be made reliably by solving this classification task using features from different time points using the sliding time anchor approach described in Section 2.6. To further adjust the complexity of the task, we select negative data sequences from patients who are infected with viruses that exhibit a particular level of CR, starting from the simplest (0CR) to the most complex (3CR); see Section 2.7 for details.

### 2.6 Sliding time anchor approach

For constructing a training dataset, we need exactly one viral sequence per patient, even though many patients have samples from multiple time points recorded in the database. An obvious choice, which we use for the baseline model, is to select the last available time point prior to developing 4CR. To study the effect of time on the capability of 4CR prediction, we additionally wish to create data sets that utilize earlier time points. However, both the frequency of time points per patient as well as their intervals are highly irregular in the data, which makes a consistent selection of earlier samples less obvious, as illustrated in Supplement S1.7.

**Figure 1.**
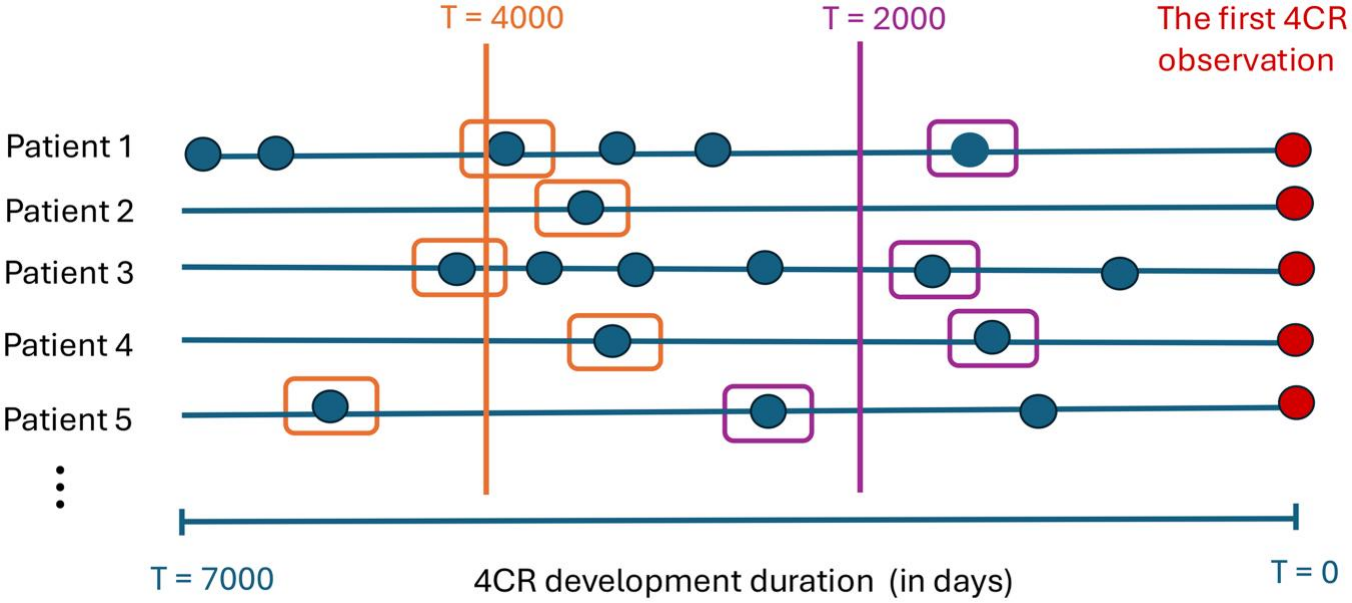
Timeline of 4CR virus development for each patient. Each anchor line represents the duration of 4CR development for individual patients. The red dot marks the initial 4CR observation, while petrol dots indicate virus sequencing events. The vertical purple line represents a sliding time anchor with T=2000 days prior to the first 4CR observation, and the vertical orange line represents another sliding time anchor with T=2000 days. Orange and purple boxes highlight the virus sequences closest to these respective time anchors.

To permit a smooth increase in the average time duration to MDR and thus the expected difficulty of the classification task, while simultaneously retaining all patients, we use a sliding time anchor approach, which is illustrated in Fig. 1. We define a time anchor T∈(0,7000) and pick, for each patient, the measurement whose time point is closest to the given sliding time anchor *T*. Hence, the extreme values 0/7000 pick the first/last available measurement for each patient, whereas other sliding time anchor values of *T* can be used to interpolate; in practice, we use increments of 1000 days.

### 2.7 Negative data representing particular CR levels

There is a significant difference in the number of patients harboring 4CR viruses compared to the patients harboring non-4CR viruses. To address this issue, we create a balanced training dataset by selecting an equal number of negative samples (patients infected with non-4CR viruses) and positive samples (patients infected with 4CR viruses).

In order to avoid bias, we select patients for the negative data who have treatment durations similar to those of the positive group. We first categorize negative patients into CR levels (0CR to 3CR). We second generate histograms of the treatment durations for each sliding anchor of positive samples. Using these histograms, we calculate the cumulative distribution function (CDF), which provides the cumulative probabilities of the treatment durations for the positive group. We randomly select histogram bins from the negative group with probabilities proportional to the positive group’s CDF. Finally, individual negative samples are then randomly drawn from the selected bins. With this approach, we ensure that the treatment duration distributions of the negative samples from the sliding time anchors are similar to those of the positive samples.

### 2.8 Mutation types

Patient virus sequences include various types of mutations that either contribute to main drug resistance or to minor drug resistance. We use not only drug-resistance mutations (DRMs) but also mutations from other categories, as they can also contribute to drug resistance. We considered the following mutation types: Major DRMs, which significantly impact drug resistance; minor DRMs, which have a weaker effect individually but can enhance the impact of major DRMs; treatment-selected mutations (TSMs), which arise under drug pressure and drive resistance; and surveillance drug-resistance mutations (SDRMs), which are useful for monitoring the transmission of drug resistance.

### 2.9 Feature extraction

The raw virus sequences from the patients have varying lengths, requiring standardization for the training dataset. Instead of using the raw sequences, we use the standard reference sequence. Due to the lack of integrase sequence data, we focus on the reverse transcriptase (RT) and protease (PR) segments. We use the 560-amino-acid length RT sequence and the 99-amino-acid length PR sequence from HXB2.

To match each patient’s virus sequence with the HXB2 reference, we substitute all recorded mutations for the RT and PR sequences at their corresponding positions. For example, if a sequence had a K65M mutation, we replaced lysine (K) with methionine (M) at position 65 in the HXB2 sequence. This process was applied to all sequences.

We use one-hot encoding to represent virus amino acid sequences, where each amino acid is encoded as a binary vector of length equal to the number of unique amino acids plus symbols (“_” and “-”) with a “1” at the position corresponding to the amino acid or symbol, with all other entries set to “0.” For ambiguous mutations, we use fractional entries that are equal to one over *m* where *m* is the number of ambiguous mutations. This method generates a vector of length 12 320 for the RT sequence, a vector of length 2178 for PR, and a final encoded vector containing a total length of 14 498 through concatenation.

As an alternative to one-hot encoding, we use the ProtT5-XL-UniRef50 Protein Language Model (Raffel *et al.* 2020, Elnaggar *et al.* 2021) to generate feature embeddings for amino acid sequences, focusing on the encoder part of the model. This model treats each amino acid as a token. Tokenizing each amino acid poses a significant computational challenge when dealing with sequences containing numerous ambiguous mutations found in patient records. To manage the computational challenge of analyzing numerous ambiguous mutations, we randomly sample and average subsets of these combinations. This approach allows us to handle up to 50 sequences with an Nvidia RTX A4000 GPU. Feature embeddings result in a vector of length 573 440 for a RT sequence, a vector of length 101 376 for a PR sequence, and by concatenating both, the combined sequence is represented by a vector of length 674 816.

## 3 Results

We developed several classification models to predict the future emergence of 4CR viruses by analyzing viral sequences extracted from both xCR levels from 0CR to 3CR, and 4CR patients. Specifically, negative samples were derived from xCR patients, while positive samples originated from 4CR patients, but all were evaluated using a sliding time anchor T=t, where t∈{0,1000,…,7000}. Importantly, even positive samples from 4CR patients were taken at times *t* when 4CR resistance has not yet emerged, ensuring the model focuses on pre-resistance signals. Therefore, we first evaluated the predictive performance by varying the classification problem using different model types, time anchors, and CR levels for negative data. Next, we also studied the feature importances from our best-performing models and related them to known drug class resistance positions and mutations listed in the Stanford HIV DB. This approach allowed us to identify predictive patterns associated with the eventual emergence of resistance to 4CR.

### 3.1 Predictive performance

We began with the baseline classification setting that distinguishes 4CR from 3CR based on the last virus sequence observed prior to 4CR with a sliding time approach T=0. Focusing on the 3CR stage was crucial because it was the most challenging type of drug class resistance to manage in a clinical setting before 4CR emergence. Patients at this stage are at high risk of progressing to 4CR. While predicting the future emergence of 4CR from 3CR was particularly difficult, using T=0 was the easiest choice.

In this setting, we built various classifiers, namely Support Vector Machines (SVMs) with RBF and polynomial kernels (Hearst *et al.* 1998), Logistic Regression (LR) with elastic net regularization (Zou and Hastie 2005), Random Forest (RF) (Breiman 2001), and XGBoost (Chen and Guestrin 2016). Since we have a balanced classification problem (284 positive and 284 negative samples), we used accuracy as the performance metric. To evaluate our models, we performed ten runs of five-fold cross-validation (CV) using the default parameters of each model from the Python Scikit-learn library.

**Figure 2.**
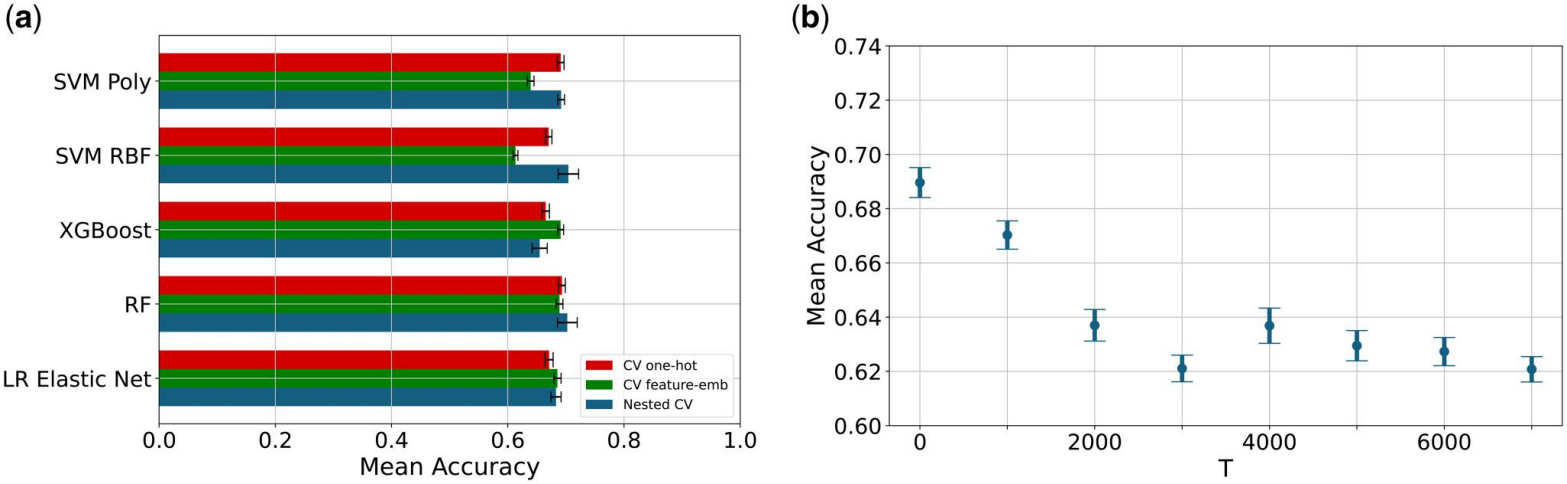
Models’ comparison for 3CR. (a) The performance evaluation across various models using CV at T=0. The red bar represents the results of CV from one-hot encoding, the green bar shows CV from feature embeddings, and the blue bar shows nested CV from one-hot encoding. (b) Random Forest performance across sliding time anchors. Error bars show the standard error of the mean.

First, we used one-hot encoding as a feature extraction strategy and observed that accuracy values were in the range of 0.665 to 0.693 (Fig. 2a, red bars). While being far from a perfect classification, these results showed that the prediction of 4CR from patients who already showed 3CR was doable to a certain degree. It was remarkable that the models differed only a little in the predictive performance; the random forest and the polynomial SVM performed both only slightly better than the other three alternatives.

Next, we investigated the effect of using feature embeddings instead of one-hot encoding (Fig. 2a, green bars). Here, we observed a drop in performance for both SVMs and the Random Forest, whereas there was a minimal improvement for the elastic net. The biggest improvement was seen for XGBoost, which did not outperform the best one, the hot encoding models, though. Overall, we concluded that feature embeddings did not substantially improve over one-hot encoding despite their potential to offer more detailed representations of virus amino acid sequences. Therefore, we used the latter for the remainder of this article.

While the previous two studies were carried out with default hyperparameters for all models, we now examined whether hyperparameter tuning can improve model performance. To this end, we applied nested cross-validation with five folds for both inner and outer loops. The tested hyperparameters within the nested CV are shown in Supplement S1.8.

We observed that all models yield a similar accuracy, ranging from 0.6549 to 0.7042 (Fig. 2a, blue bars). The SVM with an RBF kernel benefited most from the tuning; it was now on par with the Random Forest, which did slightly improve. The polynomial SVM results remained virtually unchanged, as the chosen hyperparameters were the same as the default values. Surprisingly XGboost did not benefit from the tuning at all. Overall, the effect of hyperparameter tuning was small for this classification problem on this data set.

Considering the high computational effort of a nested cross-validation for little predictive gain, we continued the study with default hyperparameters. In particular, we focused on the random forest as it was among the two best models when default hyperparameters were employed and offered, in comparison to the polynomial SVM, better interpretability due to built-in feature importance.

In all previous evaluations, we used T=0, meaning we always picked the last observation prior to 4CR for feature extraction. Then, we explored whether moving the time anchor, that is, predicting from even earlier measurements, had an effect on predictive performance. Intuitively constructing data sets with T>0 should make the classification problem harder, as virus sequences datasets from older time points might not reflect recent mutations, which can affect resistance patterns.

We tested this hypothesis for the 4CR versus 3CR classification problem using different values for *T*. The results are shown in Fig. 2b. They confirmed that the problem gets gradually harder when the time anchor is increased. The performance never degraded to complete randomness, which might seem counter-intuitive at first glance. It can be explained by the fact that even for T=7000, there were still some patients in the data set with observations relatively close to the 4CR, for instance, those that had only one additional measurement prior to 4CR in the first place.

Since each value of *T* corresponds to a selection of time points closest to that value *T*, their average time difference to the 4CR observation may be very different to *T*. Taking the extreme cases as examples, we find that T=0 corresponds to an average time gap of 1633 days, whereas T=7000 corresponds to an average time gap of 2521 days. Hence, we additionally show the mean accuracy as the function of the actual time gap in Supplement S1.9.

All evaluations described up to this point attempted to classify 4CR versus 3CR, meaning they attempted to predict the emergence of four class drug resistance in patients already exhibiting three class drug resistance. While this is arguably the clinically most relevant task, it is also the most challenging. To put the results into perspective we now also investigated the seemingly easier tasks of predicting the emergence of 4CR for the patients with the lower CR levels (0–2) versus 4CR patients at the same time anchor *T*.

**Figure 3.**
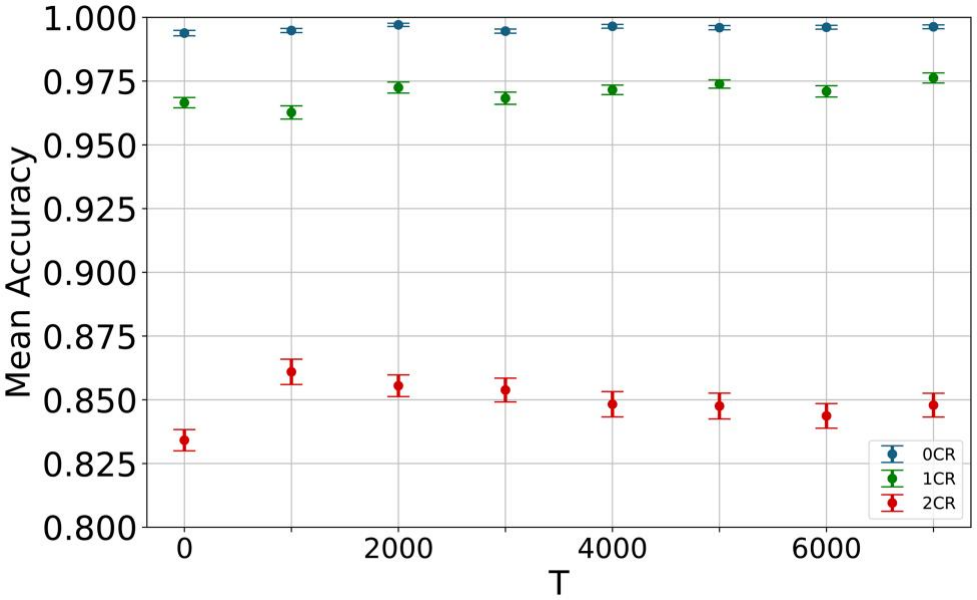
Risk of 4CR emergence. Default random forest models across sliding time anchors T∈(0,7000) approach for 0CR, 1CR, and 2CR. Error bars show the standard error of the mean.

For this purpose, we applied the same methodology as before: a default random forest learned on datasets with different time anchors *T*. The results are shown in Fig. 3. For the 0CR, we observed a nearly perfect classification for all values of *T*. This can be explained by the fact that this model essentially learned to detect any drug class resistance for those predicted with 4CR emergence in the future since the negative data did not contain any resistant viruses at all. As plenty of resistance mutations are known in the literature, it was no surprise that the ML models were also able to pick them up. The same held for the 1CR case, which distinguished MDR from single drug class resistance.

When using viral sequences of 2CR patients for the negative data, the models’ performance noticeably decreased compared to 0CR and 1CR; in terms of CR levels, it was the first problem that did not appear to be a trivial task. Yet it was, with mean accuracy scores ranging from 0.8438 and 0.8556 across all sliding sampling timelines, still a substantially easier problem than predicting 4CR emergence from the virus sequences of 3CR patients versus virus sequences of 4CR patients. This suggested that higher CR levels, reflecting greater complexity and diversity of mutations within viral sequences, negatively impacted predictive performance. Overall, these findings emphasized the importance of understanding viral sequence evolution in the context of drug class resistance, as higher CR levels presented more challenges for accurate prediction.

### 3.2 Feature importance

Next, we studied whether the trained models can accurately and reasonably learn key features related to drug resistance, identify any new or previously unrecognized features contributing to the model’s predictive accuracy, and offer new insights into drug resistance. To this end, we evaluated feature importance in terms of mean decrease in Gini impurity (MDI) from models trained on the sliding time anchor T=0 for both the 0CR and 3CR stages. We expected that the 0CR model would find the most important features associated with drug resistance by looking at its predictive performance. In contrast, the 3CR model might focus on fewer features directly related to drug resistance as it was expected that all 3CR samples contain drug resistance mutations. Due to the one-hot encoding, the models return MDI values for each pair of position and amino acid. To display these results more concisely, we aggregated all MDI values for a single position by summing over the different amino acids.

**Figure 4.**
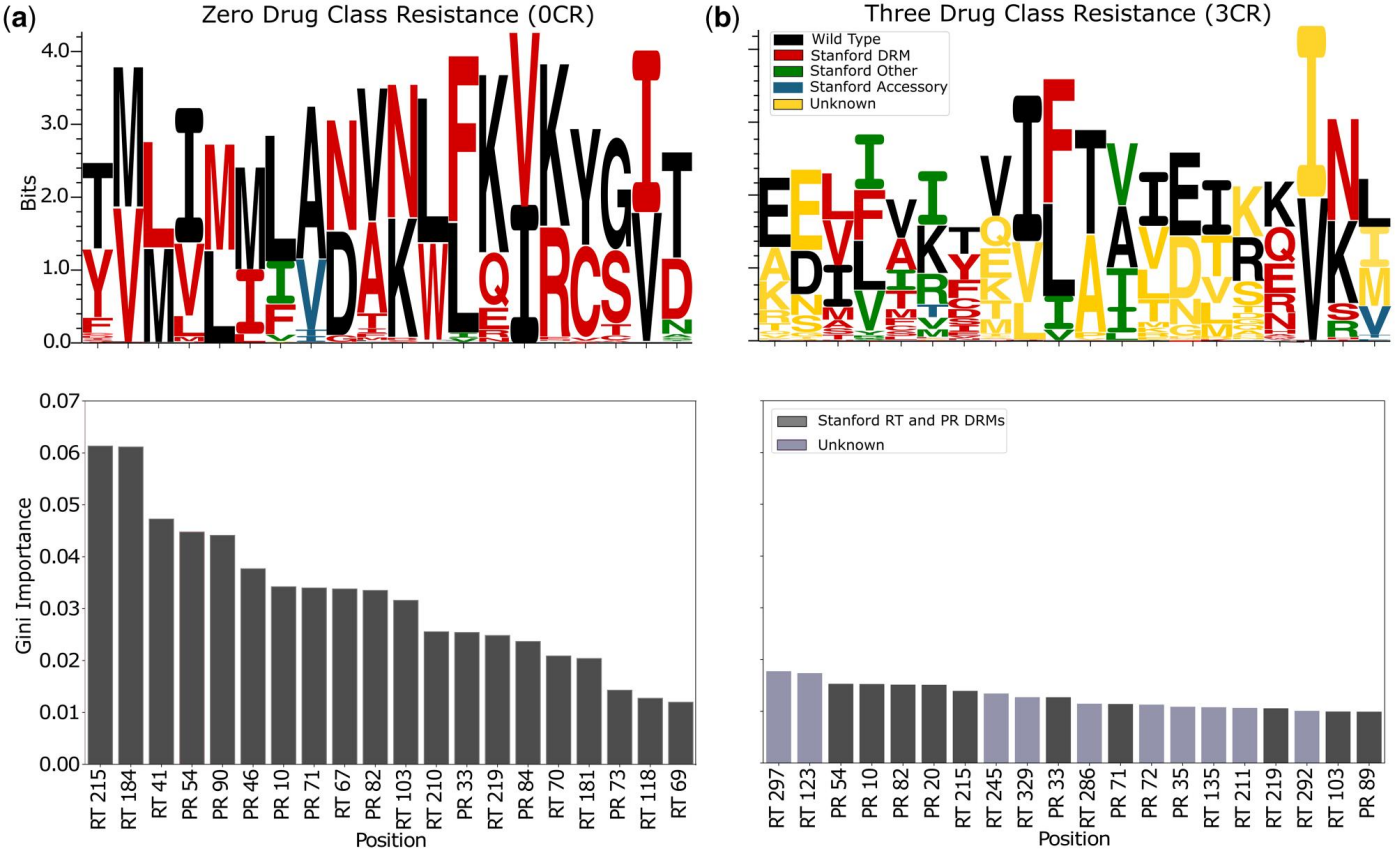
Feature importance analysis illustrating the distribution of position-specific and amino-acid-specific importance on each position for the (a) 0CR model (left) and the (b) 3CR model (right). For both-hand sides, in the lower plots, feature importance values among the top 20 ranked are color-coded based on their drug resistance mutation (DRM) category from the Stanford HIV DB: dark gray for RT and PR. If a position is within the top 20 but not listed in the Stanford DB DRM list, it is colored light gray. In the upper plots on both-hand sides, wild-type amino acids are shown in black, while mutations listed in the Stanford HIV DB as DRMs are shown in red, accessory mutations listed in the Stanford HIV DB are shown in blue, and other mutations listed in the Stanford HIV DB are shown in green. Mutations not found in the Stanford HIV DB are labeled as unknown and colored in yellow.

To check whether the top-ranked positions identified by other models were plausible, we first inspected the 0CR model, where we expected to find many known drug-resistance mutations. We plotted the top 20 most important sequence positions in decreasing order of their MDI value, ranging from 0.01 to 0.07, in the lower left side of Fig. 4. We observed that the positions RT 215 and RT 184 were the two most important positions. There was a sharp decline in feature importance following these top two positions, with the importance of the remaining positions decreasing more gradually. According to the Stanford HIV Drug Resistance DB, all of the 20 positions are known as drug resistance mutation positions in the RT or PR region. Unknown positions start to appear beyond the top 20 (see Supplement S1.10).

Next, we studied feature importance in the 3CR model to potentially identify any new, previously unidentified positions that were particularly predictive for multi drug class resistance. The aggregated MDI values per position range from 0 to 0.0175 (bottom right in Fig. 4), which is considerably lower compared to the CR case. Further, the MDI distribution across positions was more uniform. Both observations were plausible, given the lower predictive performance of the 3CR model. Half of the top 20 positions were not known as DRM positions according to the Stanford HIV Drug Resistance DB, including the two most important ones (RT 297 and RT 123). However, due to the low absolute MDI values and even distribution, no single position strikes out as particularly predictive.

While aggregating MDI values per position provided information about the location of learned drug resistance mutations, it lost information about the mutation type. To allow such a more detailed view, we normalized each original amino acid–specific MDI value by the aggregated MDI value at this position. This results in a probability distribution that represents how likely a specific amino acid is to be important and can be visualized as a sequence logo stack.

Carrying out this procedure for each of the top-20 important positions using Seq2Logo-2.0 (Thomsen and Nielsen 2012) yielded a visualization resembling a traditional sequence logo (Fig. 4, top), with three important differences. First, the sequence positions shown were not adjacent in the protein, but rather shown in order of decreasing aggregated MDI. Second, the height of each symbol was proportional to its MDI for the random forest 0CR and 3CR models, respectively, as opposed to the occurrence frequency in the sample. Third, the non-wild type amino acids were colored according to their inclusion in Stanford HIV Drug Resistance DB.

Besides known DRMs, accessory mutations either intensify drug resistance when paired with a major mutation or increase the replication fitness of viruses containing major drug resistance mutations. Mutations classified as “other” by the Stanford HIV DB were more common in patients on ART than in ART-naive individuals. Any mutations not found in the Stanford HIV DB were labeled as “unknown” to indicate that they have not yet been found to be associated with drug resistance.

In the 0CR model, the variability in amino acids across the identified positions was generally low (upper left in Fig. 4). At the most important position, RT 215, the key amino acids were T, Y, and F. At positions RT 184, RT 41, RT 103, PR 84, and RT 118, we observed a more distinct binary pattern, where two amino acids dominated, and in each case, one of these represented the wild type. In contrast, positions PR 54, PR 82, RT 219, and PR 33 exhibited greater amino acid diversity, suggesting more variation at these positions. The model primarily identified known DRMs, with a few additional accessory and other mutations. The two most critical positions, RT 215 and RT 184, contained only DRMs listed in the Stanford HIV DB. At position PR 10, different than the observed DRM pattern, the wild-type amino acid was L, with I and V classified as other mutations, and F listed as a known DRM. Similarly, for the position PR 71, A was the wild-type amino acid, while I, V, and T were accessory mutations.

In the 3CR model, the variability in amino acids at the most important sequence positions was considerably higher than in the 0CR model (upper left in Fig. 4). We observed only one position with a binary pattern, RT 292, the wild type V and precisely one mutation variant with I. It identifies previously known DRMs, accessory mutations, and other mutations. For instance, at position PR 20, T was a known accessory mutation, while K represented wild type, and I, R, V, and M represented other mutations. Furthermore, all positions unknown to the database obviously also resulted in unknown amino acids. However, there was also one known position, PR89, with unknown amino acids I and M.

## 4 Discussion

We built various ML classifiers to predict the future emergence of MDR viruses in HIV-infected patients, focusing on different drug class resistance levels and time points. By varying these factors, we assessed how early and from which drug class resistance level we can reliably predict future multidrug resistance.

To the best of our knowledge, this was the first study that used machine learning methods to predict four class drug resistance from lower resistance levels, ranging from susceptible to three-drug class resistance, in contrast to single drug class resistance or single drug resistance studies (Pironti *et al.* 2017, Steiner *et al.* 2020, Blassel *et al.* 2021, Ditz *et al.* 2023a, Paremskaia *et al.* 2023). In addition, we also varied the task’s complexity along a second dimension, namely the time span between the last clinical visit with virus sequencing and the time point at which class drug resistance occurred. Our feature importance results confirmed that many of the mutations and the positions identified were related to drug resistance. More importantly, our study uncovered unknown mutation positions and mutations such as RT E297A, E297K, E297R, D123E, D123N, and PR A71P that have not yet been labeled as related to drug resistance. This suggests that previously unlabeled or undetected mutation positions and mutations might play a role in the development of four class drug resistance.

Some of the detected mutation results discovered in our study, such as RT L228H, and PR L33F, and M41L overlapped with a recent study findings on resistance-related mutations. This study analyzed the accumulation of resistance mutations over time using mutagenetic tree models (Pirkl *et al.* 2023) showing the accumulation of mutations follows a predetermined order. Additionally, Teodoro’s study analyzed data from various patients were cross-sectionally rather than longitudinally to infer the accumulation of mutations in multidrug resistant patients (Di Teodoro *et al.* 2024). Our findings from mutations RT L10I, PR L63P, RT E44D, RT H208Y, and RT L210W overlapped with Teodoro’s and Betancor’s studies (Betancor *et al.* 2014, Di Teodoro *et al.* 2024). Both studies (Pirkl *et al.* 2023, Di Teodoro *et al.* 2024) highlighted how understanding past mutations was essential for developing future therapies and addressing drug resistance. Our study added a dynamic approach to these two previous studies by incorporating multiple class resistance levels and time points.

In the most critical clinical setting to manage (Puertas *et al.* 2020, Rossetti *et al.* 2023), a major concern arises when patients are at risk of developing multidrug class resistant viruses. These patients were already infected with three-drug class resistant viruses. Focusing on their most recent sequence data in the EIDB, we used data from the highest risk cases. The prediction results confirmed that it was the most challenging prediction task as well as the highest risk clinical challenge. The high genetic similarity between three-drug class and four-drug class resistance virus sequences made prediction harder to distinguish between three-drug class and four-drug class resistance viruses.

In the most challenging prediction task, the top 20 features had comparably low feature importance scores, with no single feature standing out. Still, the model did identify both known and previously unknown drug resistance mutations. Shifting our focus to the least challenging clinical setting, patients infected with susceptible virus sequences, the top 20 features had a higher importance score, with two features emerging as the most important. As anticipated, the top 20 features were known drug resistance mutations. This confirmed that the model can easily identify and assign higher importance scores to these key resistance mutations and making two observed mutations stand out more due to the lower complexity of susceptible virus sequences.

The further back in time, we analyzed for the most challenging prediction task and we observed a decline in model performances, suggesting that the earlier recorded sequences, being further from developing four drug class resistance, have fewer evolutionary changes or mutations. This indicated that the earlier recorded sequences may not have yet accumulated critical mutations needed to predict four drug class resistance (Pirkl *et al.* 2023). Our results underscored that predicting MDR over longer time periods became relatively more challenging.

Nevertheless, further back in time, with a time anchor at 7000 days, the model performance surprisingly did not decrease to random prediction. The reason why this happens in our study arose from the nature of the dataset, as not all patients have multiple virus sequences covering entire time intervals (or spans). We used identical time points, especially for patients with fewer virus sequence samples close to multidrug resistance time points, while selecting the closest sample to the given time anchor. When there were more virus sequences from various time points and covering a better time span in EIDB, we expected that the performance of the model for the time anchor at 7000 days might decrease to a random level.

The less critical clinical settings represented the less challenging prediction tasks, susceptible to two drug class resistance; as anticipated, we observed minimal performance difference between 0CR and 1CR models. Moving further back in time for both models, we did not observe a performance decline. This suggested that lower drug resistance levels did not indicate any significant genetic complexity over time. However, the performance of the 2CR model substantially dropped, reaching its lowest point at the closest to three-drug class resistances. This suggested that genetic complexity got higher as the virus approached a higher class resistance level. Despite this performance decrease, all these tasks remained easier tasks compared to the most challenging 3CR prediction task.

Contrary to previous studies (Ditz *et al.* 2023b, Malik and Ou 2023), the performance of the models with feature embeddings was almost the same as for the models using one-hot encoding. This suggested that while feature embeddings can be powerful in more complex datasets, in cases like ours, where the virus amino acid sequences had clear and recognizable patterns well-suited for one-hot encoding, the added complexity of feature embeddings did not improve performance.

Since there was a lack of sufficient sequence data for IN-class resistance in EIDB, we were unable to include IN resistance information in our models. This posed a challenge as we set our models’ predictions relying on NRTI, NNRTI, and PR resistance data. This gap potentially affected the performance of our models and limited a more comprehensive understanding of four drug class resistance patterns. When enough IN resistance data is available in EIDB, we expect that the models’ prediction performance will improve and have IN resistance insights into four drug class resistance development.

## 5 Conclusion

In this study, we show that predicting the future emergence of 4CR viruses is feasible using virus sequence data from different drug class resistance levels and time spans. Drug class resistance is an ongoing challenge in HIV treatment, and severe forms of drug class resistance can lead to repeated treatment failure and disease progression. The feature importance analysis shows the most critical amino acid positions and mutations, as some are known positions and mutations, while some are new. Our findings can be supportive or shed light on the early detection of four drug class resistance viruses, enable timely adjustments to treatment strategies that might delay or even prevent the progression to four drug class resistance, improve patient outcomes, and reduce the risk of advanced drug class resistance. Furthermore, our time anchor approach could be translated to other time series data sets with irregular time points.

## Supplementary Material

vbaf099_Supplementary_Data

## Data Availability

The data used for this study was obtained from the EIDB, a resource managed by the EuResist Network. Access to the EIDB is restricted and requires approval. Researchers who wish to validate or extend this study can request access to the latest version of the database by submitting an application through the EuResist Network’s partnership page: https://www.euresist.org/become-a-partner. Please note that data availability is subject to specific terms and conditions, and the EuResist Network must grant permission.

## References

[vbaf099-B1] Bekker L-G , Beyrer C, Mgodi N et al HIV infection. Nat Rev Dis Primers 2023;9:42. 37591865 10.1038/s41572-023-00452-3

[vbaf099-B2] Bertrand L , Velichkovska M, Toborek M. Cerebral vascular toxicity of antiretroviral therapy. J Neuroimmune Pharmacol 2021;16:74–89. 31209776 10.1007/s11481-019-09858-x PMC7952282

[vbaf099-B3] Betancor G , Nevot M, Mendieta J et al Molecular basis of the association of h208y and thymidine analogue resistance mutations m41l, l210w and t215y in the hiv-1 reverse transcriptase of treated patients. Antiviral Res 2014;106:42–52. 24667336 10.1016/j.antiviral.2014.03.004

[vbaf099-B4] Blassel L , Tostevin A, Villabona-Arenas CJ et al; UK HIV Drug Resistance Database. Using machine learning and big data to explore the drug resistance landscape in HIV. PLOS Comput Biol 2021;17:e1008873. 34437532 10.1371/journal.pcbi.1008873 PMC8425536

[vbaf099-B5] Breiman L. Random forests. Mach Learn 2001;45:5–32.

[vbaf099-B6] Cadosch D , Bonhoeffer S, Kouyos R. Assessing the impact of adherence to anti-retroviral therapy on treatment failure and resistance evolution in hiv. J R Soc Interface 2012;9:2309–20. 22417909 10.1098/rsif.2012.0127 PMC3405760

[vbaf099-B7] Chen T , Guestrin C. XGBoost: a scalable tree boosting system. In: *Proceedings of the 22nd ACM SIGKDD International Conference on Knowledge Discovery and Data Mining*, pp. 785–794, 2016.

[vbaf099-B8] Cohen MS , Chen YQ, McCauley M et al; HPTN 052 Study Team. Antiretroviral therapy for the prevention of hiv-1 transmission. N Engl J Med 2016;375:830–9. 27424812 10.1056/NEJMoa1600693 PMC5049503

[vbaf099-B9] Datay MI , Boulle A, Mant D et al Associations with virologic treatment failure in adults on antiretroviral therapy in South Africa. J Acquir Immune Defic Syndr 2010;54:489–95. 20395870 10.1097/QAI.0b013e3181d91788

[vbaf099-B10] Di Teodoro G , Pirkl M, Incardona F et al Incorporating temporal dynamics of mutations to enhance the prediction capability of antiretroviral therapy’s outcome for hiv-1. Bioinformatics 2024;40:btae327. 38775719 10.1093/bioinformatics/btae327 PMC11153833

[vbaf099-B11] Ditz JC , Reuter B, Pfeifer N. Inherently interpretable position-aware convolutional motif kernel networks for biological sequencing data. Sci Rep 2023a;13:17216. 37821530 10.1038/s41598-023-44175-7 PMC10567796

[vbaf099-B12] Ditz JC , Wistuba-Hamprecht J, Maier T et al Plasmofab: a benchmark to foster machine learning for plasmodium falciparum protein antigen candidate prediction. Bioinformatics 2023b;39:i86–i93. 37387133 10.1093/bioinformatics/btad206 PMC10311333

[vbaf099-B13] Elnaggar A , Heinzinger M, Dallago C et al Prottrans: towards cracking the language of life’s code through self-supervised learning. IEEE Trans Pattern Anal Mach Intell 2021;44:7112–27. 10.1109/TPAMI.2021.3095381 34232869

[vbaf099-B14] Feder A. F. , Harper K. N., Brumme C. J., Pennings P. S. Understanding patterns of hiv multi-drug resistance through models of temporal and spatial drug heterogeneity. Elife 2021;10:e69032. 34473060 10.7554/eLife.69032 PMC8412921

[vbaf099-B15] Foka FET , Mufhandu HT. Current arts, virologic failure, and implications for aids management: a systematic review. Viruses 2023;15:1732. 37632074 10.3390/v15081732 PMC10458198

[vbaf099-B16] Gupta-Wright A , Fielding K, van Oosterhout JJ et al Virological failure, hiv-1 drug resistance, and early mortality in adults admitted to hospital in Malawi: an observational cohort study. Lancet HIV 2020;7:e620–e628. 32890497 10.1016/S2352-3018(20)30172-7 PMC7487765

[vbaf099-B17] Hammer SM , Squires KE, Hughes MD et al A controlled trial of two nucleoside analogues plus indinavir in persons with human immunodeficiency virus infection and cd4 cell counts of 200 per cubic millimeter or less. N Engl J Med 1997;337:725–33. 9287227 10.1056/NEJM199709113371101

[vbaf099-B18] Hearst MA , Dumais ST, Osuna E et al Support vector machines. IEEE Intell Syst Appl 1998;13:18–28.

[vbaf099-B19] Khalilieh S , Yee KL, Sanchez R et al Clinical pharmacokinetics of the novel HIV-1 non-nucleoside reverse transcriptase inhibitor doravirine: an assessment of the effect of patient characteristics and drug-drug interactions. Clin Drug Investig 2020;40:927–46. 10.1007/s40261-020-00934-2 PMC7511279 32816220

[vbaf099-B20] Li G , Wang Y, De Clercq E. Approved HIV reverse transcriptase inhibitors in the past decade. Acta Pharm Sin B 2022;12:1567–90. 35847492 10.1016/j.apsb.2021.11.009 PMC9279714

[vbaf099-B21] Malik MS , Ou YY. Integrating pre-trained protein language model and multiple window scanning deep learning networks for accurate identification of secondary active transporters in membrane proteins. Methods 2023;220:11–20. 37871661 10.1016/j.ymeth.2023.10.008

[vbaf099-B22] McNairy ML , El-Sadr WM. Antiretroviral therapy for the prevention of HIV transmission: what will it take? Clin Infect Dis 2014;58:1003–11. 24429438 10.1093/cid/ciu018 PMC3952607

[vbaf099-B23] Owachi D , Akatukunda P, Nanyanzi DS et al Mortality and associated factors among people living with HIV admitted at a tertiary-care hospital in Uganda: a cross-sectional study. BMC Infect Dis 2024;9:24927. 10.1186/s12879-024-09112-7 PMC10885437 38388345

[vbaf099-B24] Paremskaia AI , Rudik AV, Filimonov DA et al Web service for HIV drug resistance prediction based on analysis of amino acid substitutions in main drug targets. Viruses 2023;15:2245. 38005921 10.3390/v15112245 PMC10674809

[vbaf099-B25] Peng X , Xu Y, Huang Y et al Intrapatient development of multi-class drug resistance in an individual infected with HIV-1 CRF01_AE. Infect Drug Resist 2021;14:3441–8. 34471364 10.2147/IDR.S323762 PMC8403562

[vbaf099-B26] Pirkl M , Büch J, Devaux C et al Analysis of mutational history of multidrug-resistant genotypes with a mutagenetic tree model. J Med Virol 2023;95:e28389. 36484375 10.1002/jmv.28389

[vbaf099-B27] Pironti A , Pfeifer N, Walter H et al Using drug exposure for predicting drug resistance: a data-driven genotypic interpretation tool. PLoS One 2017;12:e0174992. 28394945 10.1371/journal.pone.0174992 PMC5386274

[vbaf099-B28] Puertas MC , Ploumidis G, Ploumidis M et al Pan-resistant HIV-1 emergence in the era of integrase strand-transfer inhibitors: a case report. Lancet Microbe 2020;1:e130–e135. 35544263 10.1016/S2666-5247(20)30006-9

[vbaf099-B29] Raffel C , Shazeer N, Roberts A et al Exploring the limits of transfer learning with a unified text-to-text transformer. J Mach Learn Res 2020;21:1–67.34305477

[vbaf099-B30] Rossetti B , Incardona F, Di Teodoro G et al; EuResist Network. Cohort profile: a European multidisciplinary network for the fight against HIV drug resistance (EuResist Network). Trop Med Infect Dis 2023;8:243. 37235291 10.3390/tropicalmed8050243 PMC10222321

[vbaf099-B31] Steiner MC , Gibson KM, Crandall KA. Drug resistance prediction using deep learning techniques on HIV-1 sequence data. Viruses 2020;12:560. 32438586 10.3390/v12050560 PMC7290575

[vbaf099-B32] Tang MW , Liu TF, Shafer RW. The HIVdb system for HIV-1 genotypic resistance interpretation. Intervirology 2012;55:98–101. 22286876 10.1159/000331998 PMC7068798

[vbaf099-B33] Thomsen MCF , Nielsen M. Seq2logo: a method for construction and visualization of amino acid binding motifs and sequence profiles including sequence weighting, pseudo counts and two-sided representation of amino acid enrichment and depletion. Nucleic Acids Res 2012;40:W281–W287. 22638583 10.1093/nar/gks469 PMC3394285

[vbaf099-B34] Waters L , Mehta V, Gogtay J et al The evidence for using tenofovir disoproxil fumarate plus lamivudine as a nucleoside analogue backbone for the treatment of HIV. J Virus Erad 2021;7:100028. 33598310 10.1016/j.jve.2021.100028 PMC7868802

[vbaf099-B35] World Health Organization et al HIV drug resistance strategy. Technical report, World Health Organization, 2021.

[vbaf099-B36] Zazzi M. , Hu H., Prosperi M. The global burden of HIV-1 drug resistance in the past 20 years. PeerJ 2018;6:e4848. 29844989 10.7717/peerj.4848 PMC5971836

[vbaf099-B37] Zou H , Hastie T. Regularization and variable selection via the elastic net. J R Stat Soc Ser B Stat Method 2005;67:301–20.

