# Supplementary material for: HIV multidrug class resistance prediction with a time sliding anchor approach
Source: Bioinform Adv. 2025 May 15;5(1):vbaf099. doi: 10.1093/bioadv/vbaf099 (PMC12104520; doi:10.1093/bioadv/vbaf099)

Supplementary Material

Nurhan Arslan Ralf Eggeling Bernhard Reuter
Kristel van Leathem Marta Pingarilho Perpétua Gomes
Anders Sönnerborg Rolf Kaiser Maurizio Zazzi
Nico Pfeifer The EuResist Network Study Group

April 23, 2025

1 Drug Resistance Calculations

The Stanford HIV Drug Resistance Interpretation system is a rule-based tool that analyzes HIV-1 protease (PR), reverse transcriptase (RT), and integrase (IN) sequences submitted by users to assess resistance levels to 24 FDA-approved antiretroviral drugs. Each mutation, along with certain combinations of mutations associated with drug resistance, is assigned a specific penalty score. These individual scores are summed to produce a total resistance score, or "rscore," for each drug within the optimized background therapy. Based on the rscore, drug resistance is classified into five levels. Level 1 (susceptible) corresponds to an rscore below 10, indicating no reduction in susceptibility compared to the wild-type virus. Level 2 (potential low-level resistance), with an rscore between 10 and 15, reflects mutations often linked to prior drug exposure or found alongside other resistance mutations, but not strongly indicative of resistance on their own. Level 3 (low-level resistance), assigned to rscores between 15 and 30, represents mutations that reduce drug susceptibility in vitro or are associated with suboptimal treatment outcomes. Level 4 (intermediate resistance), with an rscore between 30 and 60, suggests reduced drug efficacy, though the drug is still expected to retain significant antiviral activity. Finally, Level 5 (high-level resistance), marked by an rscore of 60 or above, indicates a substantial loss of drug effectiveness due to mutations that confer resistance similar to that seen in highly resistant viral strains.

1.1 Drugs in Drug Classes

We specifically examine the following drugs within each drug class. NRTI drug class includes Abacavir (ABC), Lamivudine (3TC), Emtricitabine (FTC), Tenofovir (TDF or TAF), Zidovudine (AZT or ZDV) (other drugs in the class such as Didanosine, Stavudine, Zalcitabine are excluded because they are no longer in use.) NNRTI class includes Nevirapine (NVP), Efavirenz (EFV), Etravirine (ETR), Rilpivirine (RPV), Doravirine (DOR) (other drugs in the class such as Delavirdine are excluded because they are no longer in use.) PI drug class includes Lopinavir (LPV), Atazanavir (ATV), Darunavir (DRV) (other drugs in the class such as Saquinavir, Indinavir, Ritonavir, Amprenavir, Fosamprenavir, Tipranavir are excluded because they are no longer in use.). IN drug class contains Raltegravir (RAL), Elvitegravir (EVG), Dolutegravir (DTG), Bictegravir (BIC).

1.2 Alternative IN resistance definition

This definition suggests that IN-based therapy may result in emergent resistance to INs under specific conditions as follows: any RAL or EVG treatment with [two consecutive viral loads (VL = HIV-1 RNA copies/ml) > 50 OR one viral load > 1000] after four months of continuous treatment with the same RAL or EVG (changes in companion drugs are allowed). Any DTG or BIC treatment that includes [2 consecutive viral loads (VL = HIV-1 RNA copies/ml) > 50 OR 1 VL > 1000] following the completion of at least 4 months of continuous treatment with the same DTG or BIC (changes in companion drugs are permitted), except for patients receiving their very first treatment with DTG or BIC (this exception is based on the fact that emergent resistance to INs does not result from the failure of first-line DTG or BIC). Whenever one of these circumstances occurs, the date of the second VL of the consecutive pair > 50 or the date of the single VL > 1000 is used to determine if the patient is IN resistant.

1.3 Drug Resistance

The drug resistance results for each drug class are shown below.

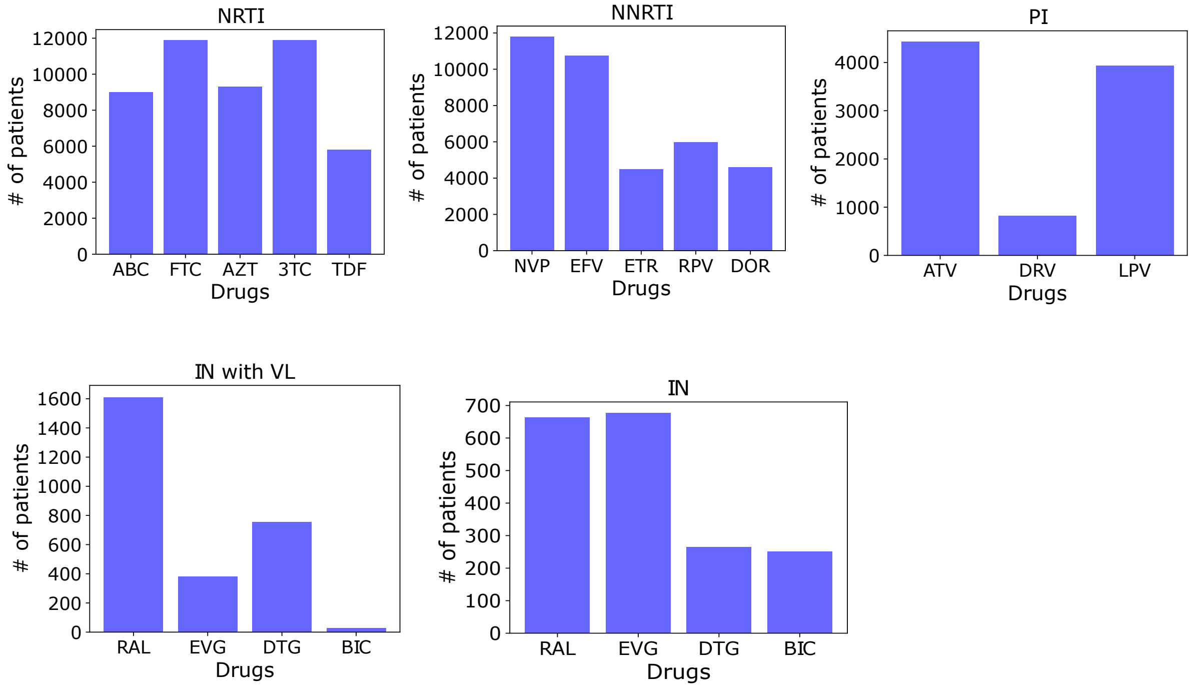

1.4 Illustration of Drug Class Resistance Classification

The illustration of CR classification is presented in figure[1] below. It shows the process of labeling patients infected with viruses at different CR levels. The diagram starts by depicting drug resistance as the foundation, followed by an explanation of each CR level. It also highlights how negative and positive samples are categorized according to these definitions.

[1]Arslan, N. (2025). *Illustration of Drug Class Resistance Classification.* Created in BioRender. https://BioRender.com/k94u663

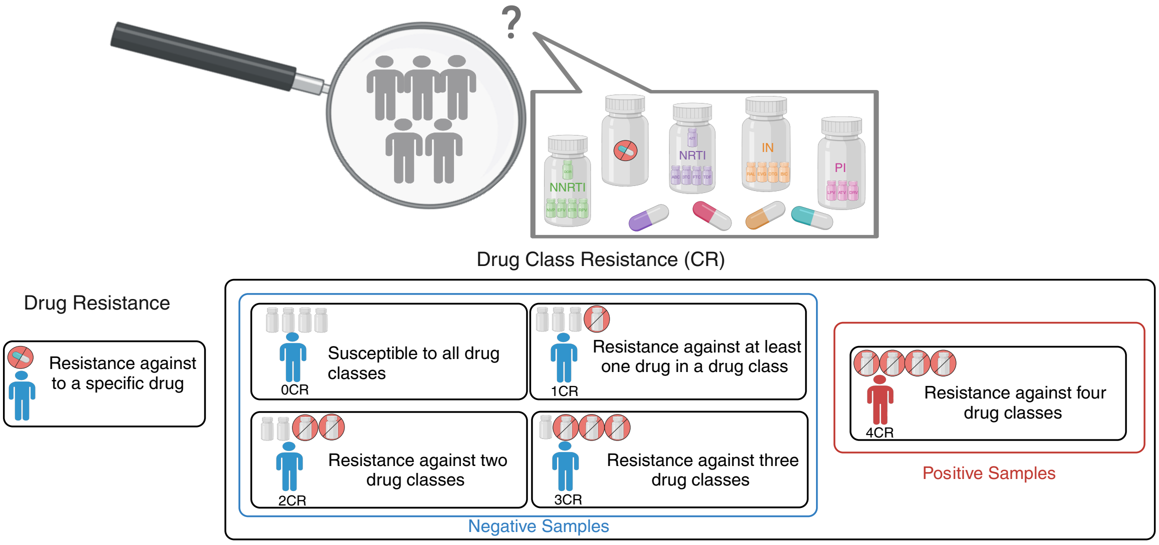

1.5 Analyzing the Distribution of Patients Resistant to Specific Drug Classes and CR levels

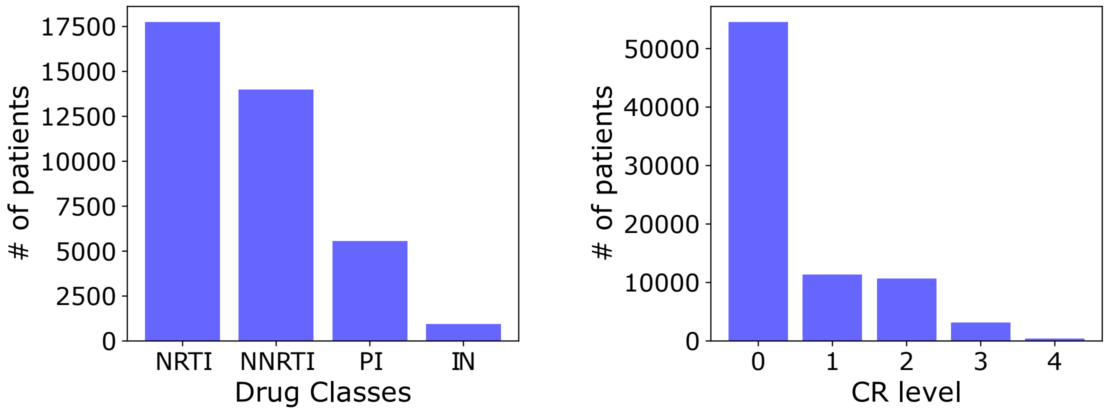

1.6 Histogram Sampling

We compare the duration of 4CR development in positive patients to the treatment duration in negative patients. For positive patients, we calculate the 4CR development time as the interval between the first virus sequencing event and the first sequencing event that detects 4CR in the database. For negative patients, we determine treatment duration from their first to their most recent virus sequencing event. As shown in the figure below, there is a noticeable difference in duration between the two groups. To address this, we use histogram sampling to select a subset of negative patients whose treatment timelines closely match those of positive patients.

We begin by defining a treatment duration range for negative patients that matches the shortest and longest 4CR development times observed in positive patients. We then group negative patients according to CR levels from 0CR to 3CR. For each CR level, we calculate the cumulative distribution function (CDF) of treatment durations in positive patients and generate a corresponding number of random numbers. We map these random

numbers to the bins of the CDF. We determine the frequency of occurrences in each bin and assign negative patients to the corresponding bins based on their treatment duration. Then, we randomly sample a number of unique negative patients equal to the number of positive cases. This procedure is repeated for each CR level and its corresponding time anchors. The outcomes of the histogram sampling are illustrated in the figure below.

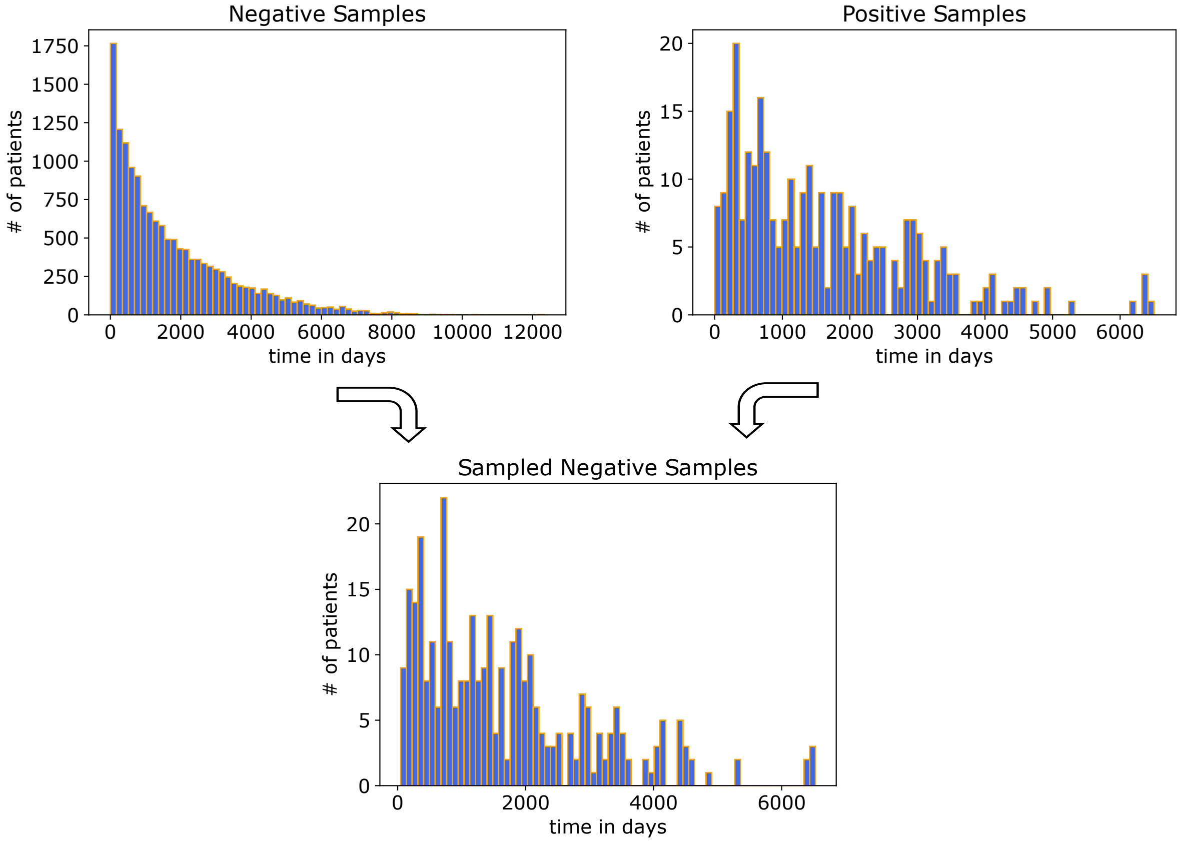

The top-left figure shows the under-treatment durations for negative patients, including all negative patients across different CR levels. The top-right figure displays the distribution of 4CR development durations for positive patients. The bottom figure presents a sampled subset of negative patients with 3CR, whose under-treatment durations align with those of positive patients at the time anchor $T = 0$.

By using this method, we ensure that both groups experience equal under-treatment durations. Negative patients with treatment durations similar to positive patients are at a higher risk of developing resistance, as prolonged ART use can exert selective pressure on the virus, potentially leading to adaptation and reduced treatment effectiveness.

1.7 Sliding Time Anchor Approach

The following figure represents all virus sequencing events from multiple time points for each positive patient recorded in the DB. 0 in time shows the initial 4CR observation, while each dot indicates virus sequencing events. Red dots represent the closest sequencing event to the initial 4CR observation for each patient.For each time anchor, we select a virus sequencing event from each patient that is closest to the given time anchor.

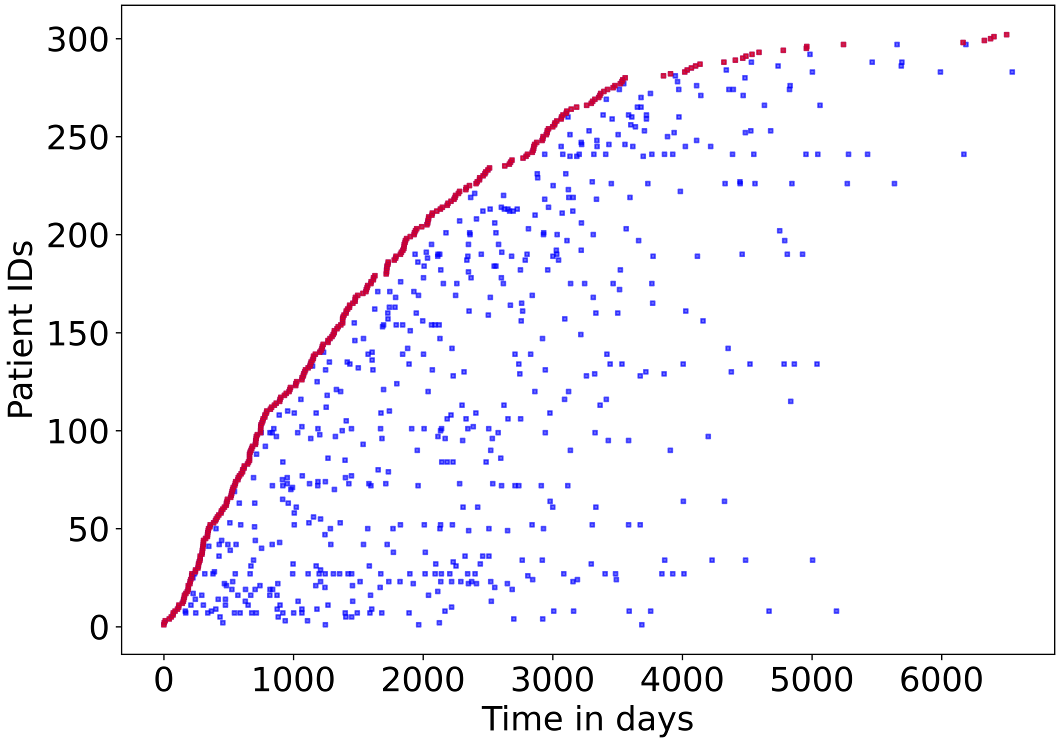

1.8 Hyperparameter Tuning

The table below presents the hyperparameter ranges for various classifiers. Each classifier has specific tuning parameters that are explored within these defined ranges to optimize model performance.

Classifier	Parameter 1	Ranges
RF	n_estimators<br>max_features	1, 100, 300, 500, 700, 900, 1000, 2000, 3000<br>1, log2, sqrt, 400, 1000, 5000, 14498
SVM Polynomial	C<br>Degree	0.01, 0.1, 1, 10, 100, 1e3, 1e4, 1e5<br>1, 2, 3, 4, 5, 6, 7, 8, 9
SVM RBF	C<br>Gamma	0.001, 0.01, 0.1, 1, 10, 100<br>1e-4, 0.001, 0.001595176, 0.01, 0.1, 1, 10, 100
XGBoost	n_estimators<br>learning_rate	1, 100, 300, 500, 700, 1000<br>0.0001, 0.001, 0.01, 0.1, 0.3

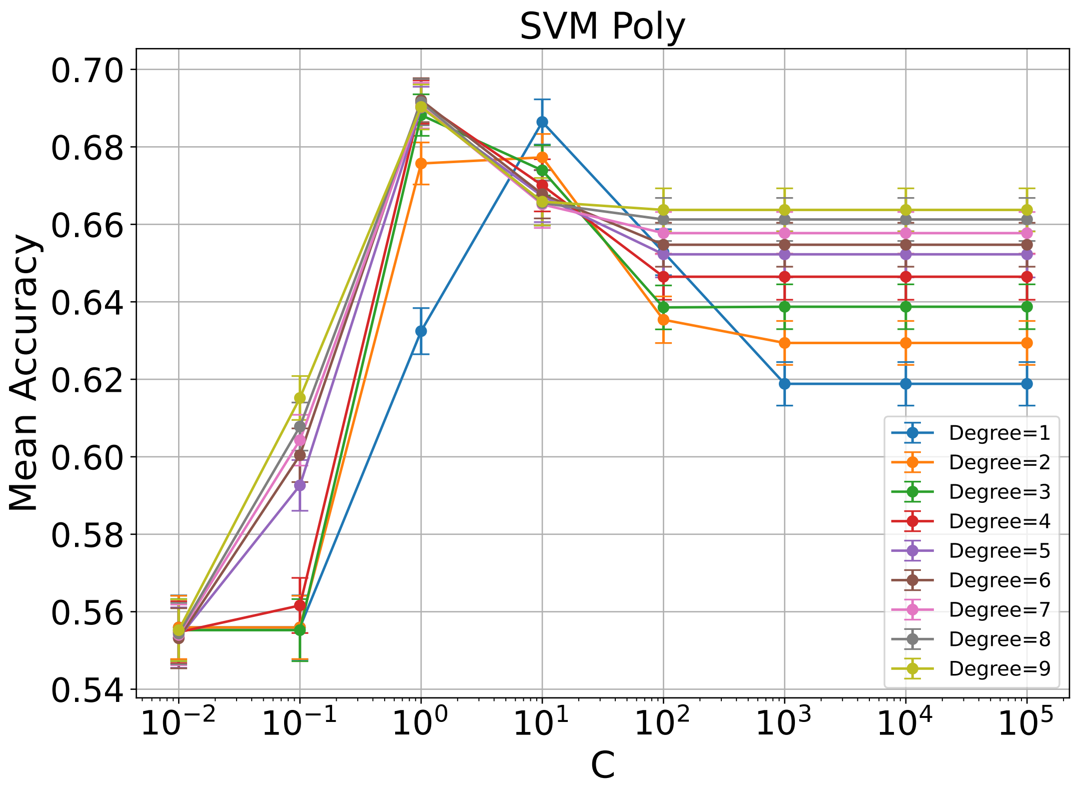
SVM Poly
Mean Accuracy
C
0.54
0.56
0.58
0.60
0.62
0.64
0.66
0.68
0.70
10^{-2}
10^{-1}
10^{0}
10^{1}
10^{2}
10^{3}
10^{4}
10^{5}
Degree=1
Degree=2
Degree=3
Degree=4
Degree=5
Degree=6
Degree=7
Degree=8
Degree=9

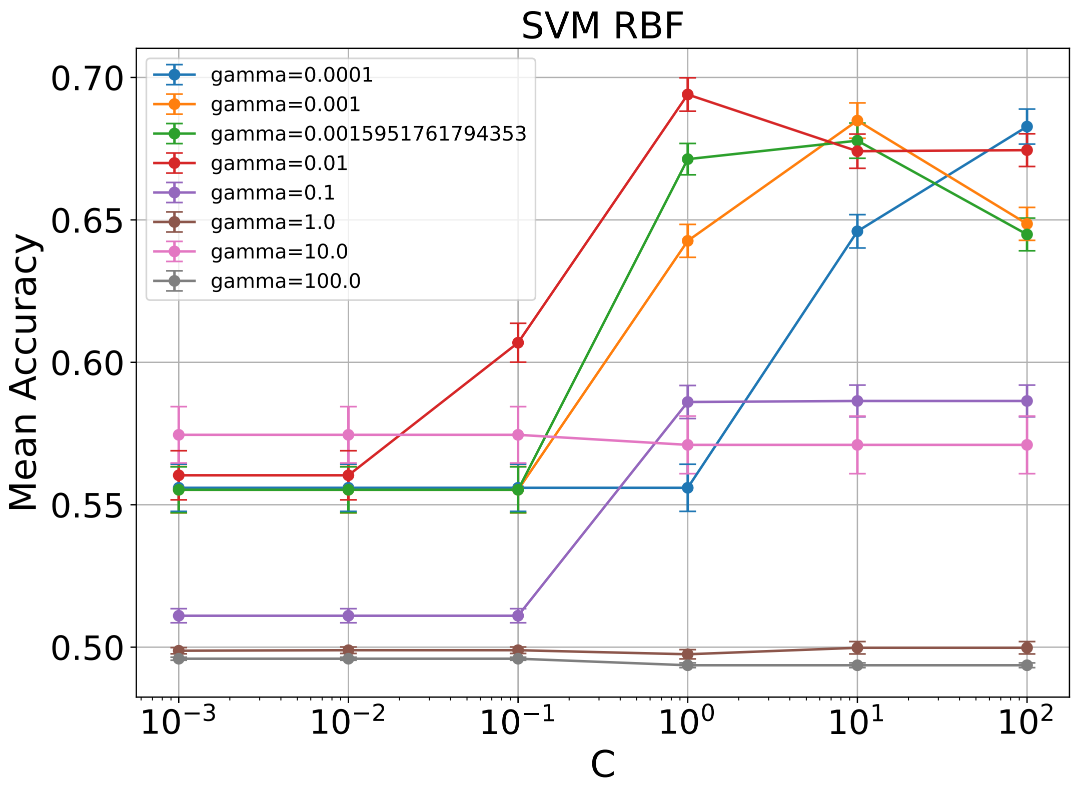
SVM RBF
Mean Accuracy
C
0.50
0.55
0.60
0.65
0.70
10^{-3}
10^{-2}
10^{-1}
10^{0}
10^{1}
10^{2}
gamma=0.0001
gamma=0.001
gamma=0.0015951761794353
gamma=0.01
gamma=0.1
gamma=1.0
gamma=10.0
gamma=100.0

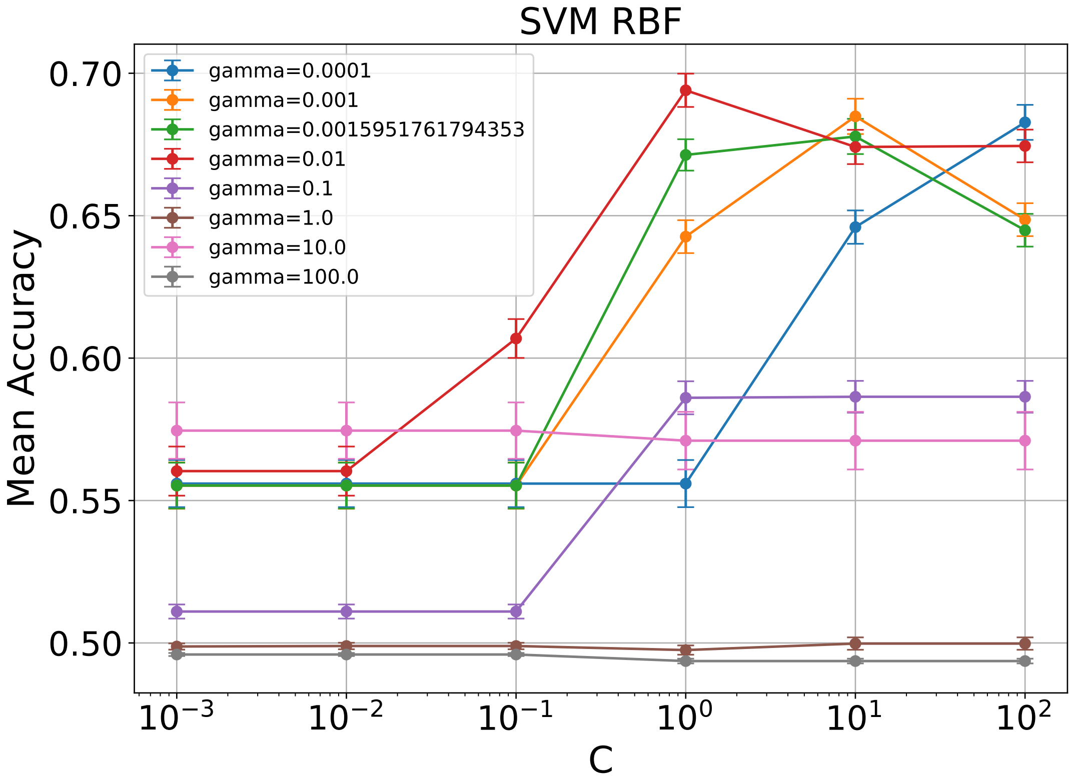

SVM RBF
gamma=0.0001
gamma=0.001
gamma=0.0015951761794353
gamma=0.01
gamma=0.1
gamma=1.0
gamma=10.0
gamma=100.0
Mean Accuracy
0.70
0.65
0.60
0.55
0.50
10^{-3}
10^{-2}
10^{-1}
10^{0}
10^{1}
10^{2}
C

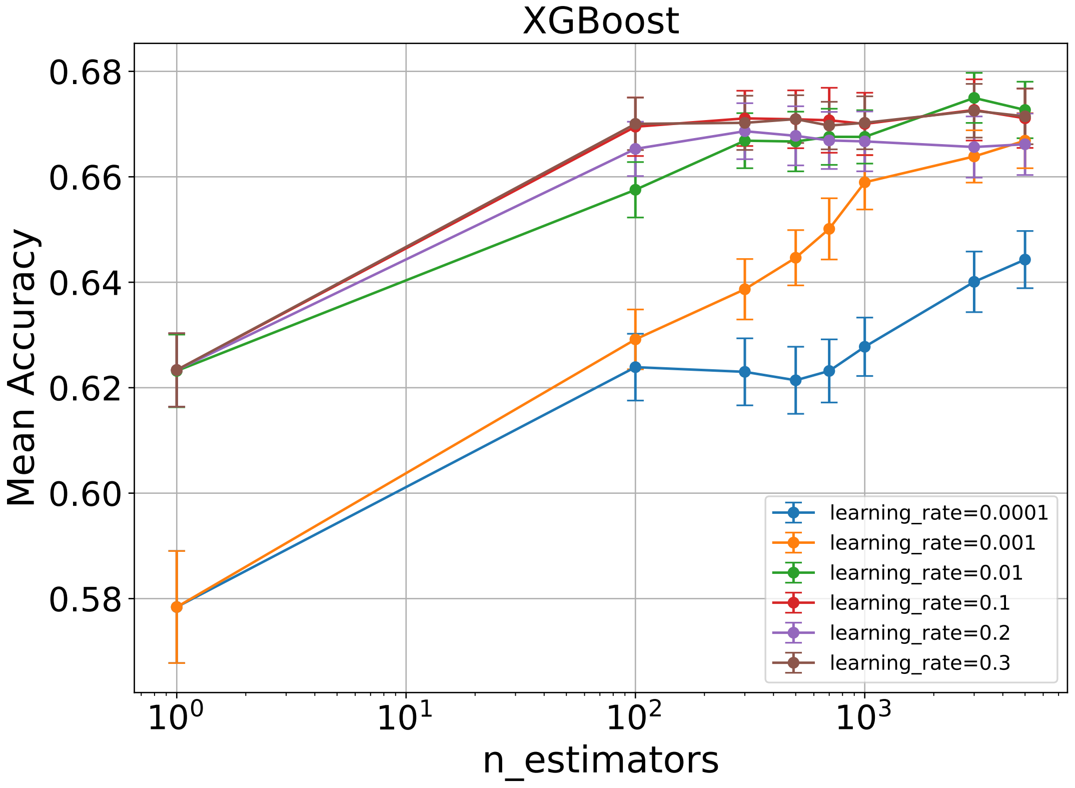

XGBoost
Mean Accuracy
0.68
0.66
0.64
0.62
0.60
0.58
learning_rate=0.0001
learning_rate=0.001
learning_rate=0.01
learning_rate=0.1
learning_rate=0.2
learning_rate=0.3
10^{0}
10^{1}
10^{2}
10^{3}
n_estimators

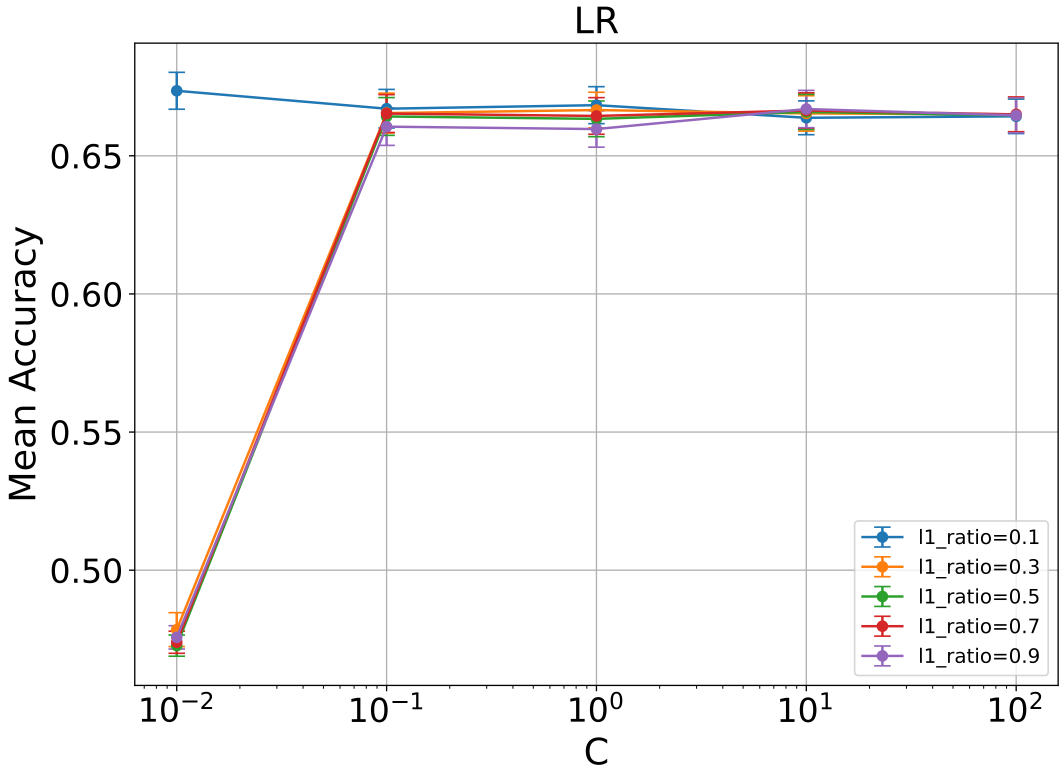

1.9 RF results across the average time gaps

The figure above shows RF performance across across the average time gaps for the 3CR model.

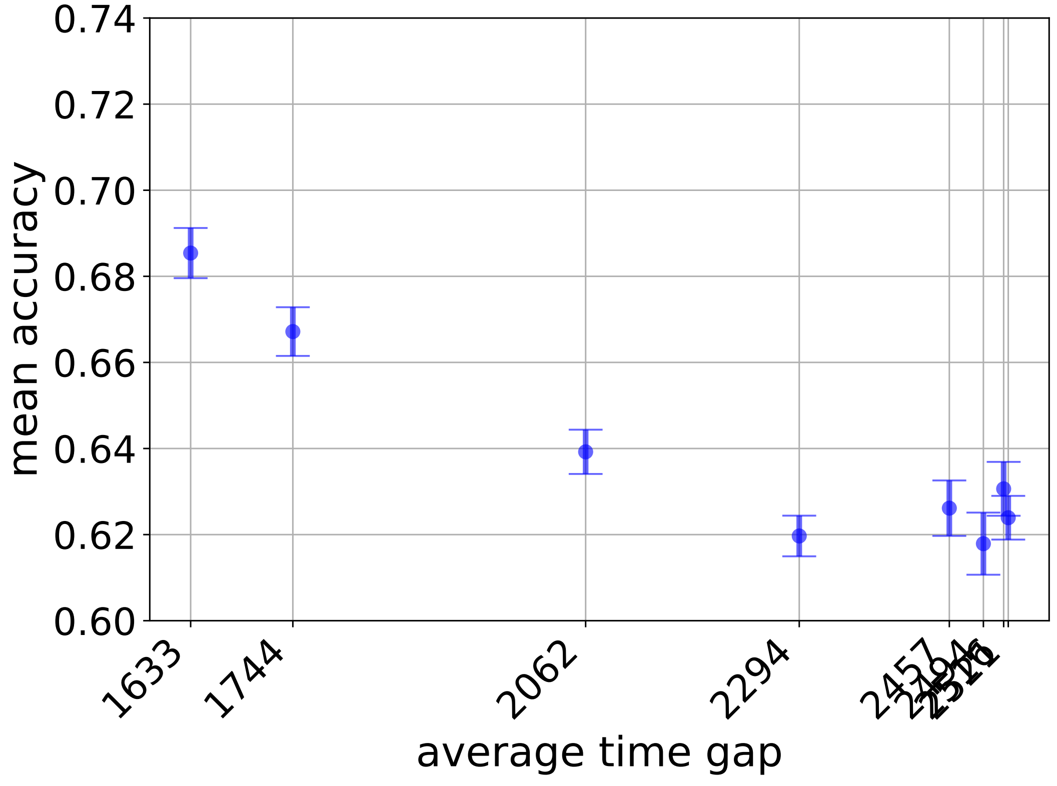

1.10 Feature Importance

The following figure shows the distribution of position specific feature importance for 0CR model at time anchor $T = 0$.

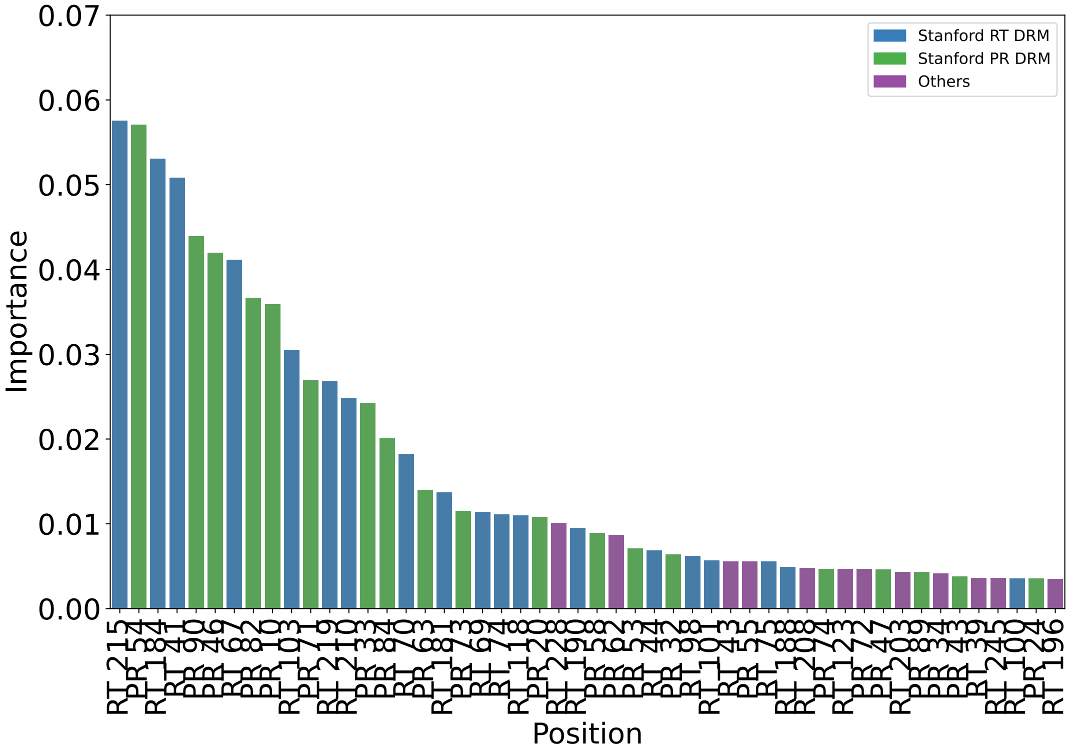

The figure below illustrates the distribution of position specific feature importance for 3CR model at time anchor $T = 0$.

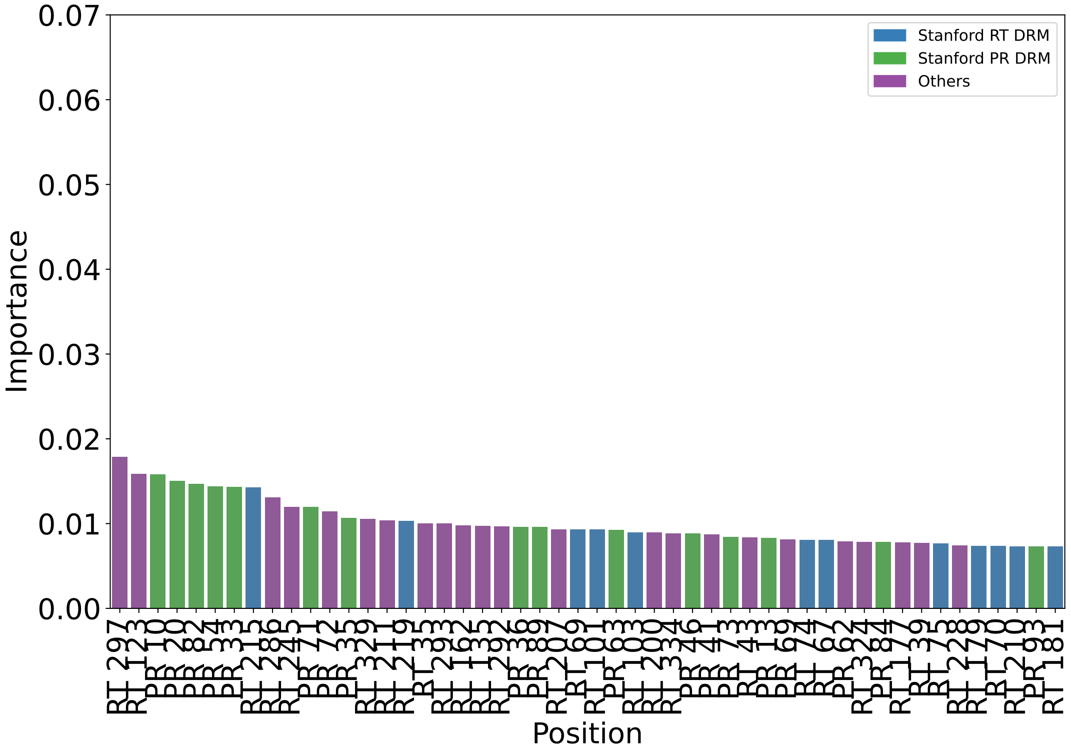

Supplement: vbaf099_Supplementary_Data [file vbaf099_supplementary_data.pdf]
